# Management of Bleeding Events Associated with Antiplatelet Therapy: Evidence, Uncertainties and Pitfalls

**DOI:** 10.3390/jcm9072318

**Published:** 2020-07-21

**Authors:** Anne Godier, Pierre Albaladejo, the French Working Group on Perioperative Haemostasis (GIHP)

**Affiliations:** 1 Department of Anesthesia and Intensive Care, GEGP-AP-HP, 75015 Paris, France; 2 Department of Anesthesia and Intensive Care, Grenoble University Hospital, 38700 La Tronche, France; PAlbaladejo@chu-grenoble.fr

**Keywords:** bleeding, antiplatelet agents, platelet transfusion, platelet function test, guidelines

## Abstract

Bleeding complications are common in patients treated with antiplatelet agents (APA), but their management relies on poor evidence. Therefore, practical guidelines and guidance documents are mainly based on expert opinion. The French Working Group on Perioperative Haemostasis provided proposals in 2018 to enhance clinical decisions regarding the management of APA-treated patients with a bleeding event. In light of these proposals, this review discusses the evidence and uncertainties of the management of patients with a bleeding event while on antiplatelet therapy. Platelet transfusion is the main option as an attempt to neutralise the effect of APA on primary haemostasis. Nevertheless, efficacy of platelet transfusion to mitigate clinical consequences of bleeding in patients treated with APA depends on the type of antiplatelet therapy, the time from the last intake, the mechanism (spontaneous versus traumatic) and site of bleeding and the criteria of efficacy (in vitro, in vivo). Specific antidotes for APA neutralisation are needed, especially for ticagrelor, but are not available yet. Despite the amount of information that platelet function tests are expected to give, little data support the clinical benefit of using such tests for the management of bleeding events in patients treated or potentially treated with APA.

## 1. Introduction

The four main oral antiplatelet agents (APA) have two different platelet molecular targets: aspirin inhibits the enzyme cyclooxygenase 1 and consequently thromboxane A_2_ synthesis, while clopidogrel and prasugrel (two thienopyridines), and ticagrelor inhibit the adenosine diphosphate (ADP) pathway via the receptor P2Y_12_. Whereas these drugs have demonstrated benefits regarding the prevention of arterial thrombosis and especially the recurrence of thrombotic events within appropriate indications, their use may result in bleeding complications. Although bleeding is frequent in patients treated with APAs, its treatment is poorly codified. As solid clinical evidence is lacking, practical guidelines and guidance documents are mainly based on expert opinion. The French Working Group on Perioperative Haemostasis (GIHP) and the French Study Group on Thrombosis and Haemostasis (GFHT) provided proposals in 2018 to support clinical decision regarding the treatment of APA-treated patients in emergency settings [1]. In light of the GIHP guide for practice, this brief review will discuss the evidence and uncertainties of the management of patients with a bleeding event while on antiplatelet therapy, including the means to neutralise APAs, their potential clinical benefit and risks, and the place of platelet function testing (PFT). The GIHP proposals are presented in italics.

## 2. Means to Neutralise APAs

The rationale for platelet transfusion to neutralise APA effects on haemostasis during bleeding is to provide platelets that have not been exposed to APAs and restore primary haemostasis in spite of the presence of patient’s platelets inhibited by APAs in the circulating blood. However, platelet transfusion neutralises APA only after most of the active compounds have been cleared from the circulation. Aspirin and thienopyridines are irreversible platelet inhibitors, their active compounds are shortly present in blood (15–20 min, 30 min and 4 h, for aspirin, clopidogrel, and prasugrel, respectively) [1], thus their effects on platelet aggregation can be readily neutralised by platelet transfusion after the active metabolites have been cleared. Practically, *the type of APA and the time of the last intake should be noted, in order to take into account the presence of circulating active metabolites* [1]*. In situations requiring neutralisation of aspirin, platelet transfusion should be administered at a dose of 0.5 to 0.7 × 10^11^ per 10 kg of body weight. A higher dose (double or higher) is proposed in patients treated with clopidogrel or prasugrel. The efficacy of platelet transfusion can be reduced if the time interval since the last intake of clopidogrel or prasugrel is less than six hours. Tranexamic acid should be administered owing to its ability to reduce bleeding whether the patient has received APA or not.* Of note, the definition of ‘platelet unit’ in available reports is confusing. On the one hand, since a single donation of whole blood by one donor enables the recovery of roughly 0.5 × 10^11^ platelets, such a number of platelets often, but not always, represents the platelet unit (see blood products ratios for massive transfusion). On the other hand, one platelet concentrate for transfusion in adults, whether made of pooled platelets from whole blood donations of several donors or obtained with apheresis from one single donor, can also misleadingly be named a ‘platelet unit’, whatever its actual platelet content [1]. The nation-based regulations state that a platelet concentrate contains a minimal number of platelets (2 to 2.5 × 10^11^ platelets).

Neutralisation of ticagrelor is challenging. First, platelet transfusion is ineffective to neutralise ticagrelor in contrast to other APAs. Unlike the thienopyridines, ticagrelor is a directly active P2Y_12_ inhibitor and does not require metabolic activation. Unbound plasma concentrations of ticagrelor and its first active metabolite, which is also a platelet inhibitor, are high. Although their effects are reversible, their half-lives are long: 7 and 8.5 h for ticagrelor and its active metabolite, respectively [1]. Therefore, circulating ticagrelor and its first metabolite can inhibit platelets provided by transfusion [2,3,4] for up to 24 h after the last intake [5]. In vitro or ex vivo non-inhibited platelet supplementation was shown to be unable to correct ADP-induced platelet aggregation inhibited by ticagrelor [2,3]. ADP responsiveness of donor platelets was dramatically reduced by even low (10%) concentrations of plasma prepared from ticagrelor-treated patients [6]. Considering the elimination half-lives of ticagrelor and its first metabolite, Kruger et al. extrapolated from in vitro results the appropriate quantity of transfused platelets and timing since the last dose of ticagrelor to restore platelet aggregation [7]. They suggested that the transfusion six apheresis concentrates of donor platelets might produce 90% reversal at 24 h after the last dose of ticagrelor. Nevertheless, in another study, ex vivo addition of platelets from a concentrate did not improve ADP-induced aggregation, even several days after ticagrelor discontinuation [8]. Likewise, transfusion of 8.5 × 10^11^ platelets to a patient requiring urgent neurosurgery 28 h after the last administration of ticagrelor combined with aspirin increased platelet count but did not improve ADP-induced aggregation evaluated with VerifyNow^®^ [4]. Finally, 52 patients were transfused (about 3.5 × 10^11^ platelets) prior to coronary artery bypass surgery because they had been treated with aspirin and clopidogrel (*n* = 45), prasugrel (*n* = 6), or ticagrelor (*n* = 3) and presented active bleeding. Platelet function testing revealed significant improvement of platelet function after transfusion in patients treated with clopidogrel, while there was no effect in those treated with ticagrelor (and prasugrel as well) [9]. Hence, *in situations requiring neutralisation of ticagrelor and when the time-interval since the last intake is less than 24 h, no specific treatment can be proposed because platelet transfusion at the doses used to neutralise other APAs will be ineffective. The clinical efficacy of higher doses of transfused platelets has not been evaluated. When the time-interval since the last intake of ticagrelor is greater than 24 h, platelet transfusion could provide partial recovery.* Other therapeutic options, such as desmopressin and recombinant activated factor VII (rFVIIa), have been considered, but the reported results are disappointing. Administration of desmopressin to 21 healthy volunteers treated with ticagrelor did not improve platelet function and did not reduce the bleeding time [10]. Therefore, according to the GIHP, *administration of desmopressin to neutralise APAs is not proposed*. rFVIIa, proposed by ticagrelor SmPC, is supported with limited data: the injection of rFVIIa in ticagrelor-treated mice decreased blood loss and bleeding duration after tail cut [11]. On the other hand, rFVIIa did not reduce bleeding in rabbits treated with clopidogrel [12] or with prasugrel, while rFVIIa was associated with more arterial thrombotic events [13]. Sorbent hemadsorption has also been proposed for ticagrelor removal from blood [14] . In vitro experiments using human blood mixed with ticagrelor showed an excellent capacity of sorbents to bind and remove ticagrelor (99% in plasma and whole blood) but this approach is time consuming as it requires several hours, which might be too long in case of severe bleeding.

MEDI2452, a specific antidote for ticagrelor, is in advanced development. MEDI2452 binds to circulating ticagrelor and ticagrelor-active metabolite with an affinity 100-fold higher than the affinity of ticagrelor for the P2Y_12_ receptor [15,16]. After promising results in animal studies suggesting efficacy and safety, this antidote provided immediate and sustained reversal of the antiplatelet effects of ticagrelor in healthy volunteers, as measured by multiple assays [17]. However, this neutralisation strategy is not yet available for clinical use.

## 3. Management of Bleeding Associated with APAs 

**Figure 1:**
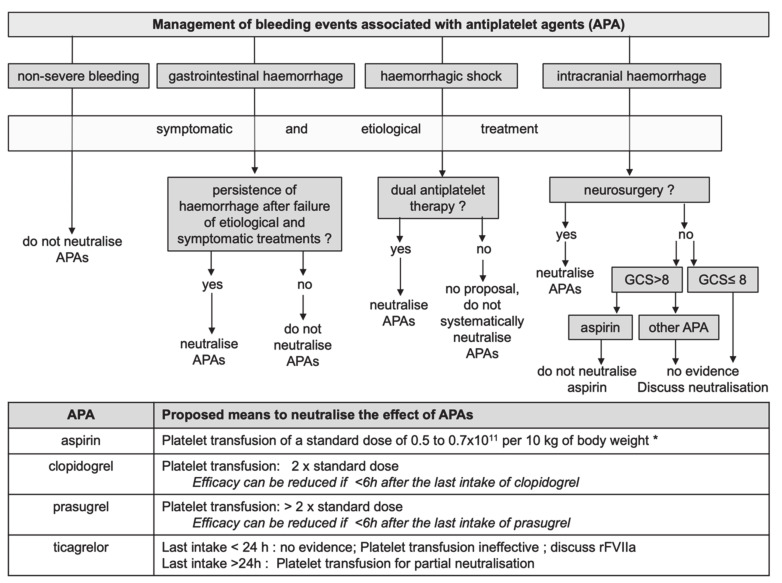
Management of bleeding events associated with antiplatelet therapy.

As stated in most guidance documents, the decision to reverse antiplatelet therapy depends on the type of APA, the time of the last intake, the thrombotic risk of the patient, as well as the characteristics of the bleeding event (site, severity) and the conventional haemostatic means (embolisation, endoscopy, surgery, etc.) (Figure 1). The use of any specific reversal of antithrombotic therapies (platelet transfusion or other in the case of APA) has to be considered only when all non-specific means have been implemented. This includes mechanical means to stop bleeding (surgery, embolisation, endoscopy, compression, etc.) and resuscitation manoeuvres such as prevention of hypothermia, fluids, vasopressors, red blood cells, plasma, or factor concentrates. Early administration of tranexamic acid is essential and recommended in cases of severe bleeding [18,19]. The efficacy and safety of this inexpensive antifibrinolytic agent have been evaluated in large trials in traumatology, post-partum haemorrhage, or in cardiac surgery (for APA-treated patients). Tranexamic acid reduced bleeding and was not associated with more thrombotic events [20]. Of note, bleeding events are a thrombotic risk factor for patients treated with APAs. This excess risk of myocardial infarction and major cardiac events has been reported for coronary stented patients in the perioperative setting [21] or after haemorrhage in other context [22].

Three clinical situations need to be addressed due to their frequency and/or severity: haemorrhagic shock, intracranial bleeding, and gastrointestinal bleeding.

### 3.1. Haemorrhagic Shock

Management of haemorrhagic shock relies on both pathophysiology and evidence-based approaches. Restoring platelet competency in this situation is considered essential and critical, though not really evaluated. Hence, neutralisation of APA will be based on the assumption that the type of APA treatment (particularly dual antiplatelet therapy (DAPT) or the new P2Y_12_ inhibitors) is associated with an increased risk of bleeding. As a result, most guidelines and guidance documents propose to neutralise APA, although the benefit of neutralisation in this situation has not been evaluated [1,19]. On the other hand, early platelet transfusion is recommended to treat haemorrhagic shock in attitudes based on high plasma/platelet/packed red blood cells ratios, independently of any chronic treatment interfering with haemostasis [19]. 

In severe trauma patients, the excess risk of bleeding caused by APA is not well established. In elderly severe trauma patients, pre-injury APA was associated with neither the amount of packed red blood cells nor the need for massive transfusion [23]. In another cohort of trauma patients, ongoing antiplatelet therapy did not significantly increase the risk of mortality [24]. Nevertheless, in a recent large cohort of severe trauma patients, preinjury antiplatelet therapy was one of the most important risk factors to predict the need for massive transfusion [25]. Therefore, despite the lack of evidence, APA neutralisation is usually proposed.

### 3.2. Intracranial Haemorrhages (ICH)

Intracranial haemorrhage (ICH) is an event frequently observed (10% to 30%) in patients chronically treated with APA [26]. Nevertheless, the relationship between antiplatelet therapy and intracranial haemorrhage is controversial: whereas we used to believe that antiplatelet therapy was a major risk factor for intracranial haemorrhage, strong evidence shows that, in fact, aspirin is not associated with an increased incidence of intracranial haemorrhage [27,28]. Similarly, there are increasing data suggesting that P2Y_12_ inhibitors do not increase this risk [29,30,31]. Nevertheless, once intracranial haemorrhage occurs, antiplatelet therapy worsens the prognosis. The excess of mortality increases gradually and seems to be related to the APA regimen. In patients on DAPT, the mortality is higher than mortality of patients treated with aspirin alone [32], which is also higher than untreated patients [33]. However, relationship between APA and mortality is still discussed. Indeed, among patients with ICH, previous use of DAPT, but not single APA, was associated with higher risk for in-hospital mortality [34]. Clopidogrel is also independently associated with mortality [33]. Early transfusion of platelets in patients with spontaneous ICH theoretically aimed to reduce volume expansion of a haematoma or the amount of bleeding and thereby improve prognosis. In an observational study of patients presenting intracranial haemorrhage during treatment with aspirin, early platelet transfusion within 12 h of symptom onset compared with late platelet transfusion after the 12th hour was associated with less haematoma expansion and a more limited disability at three months [35]. Platelet transfusion given before cranial decompressive surgery was partially effective in patients on clopidogrel [36]. A meta-analysis of studies (observational studies) evaluating platelet transfusion in patients on antiplatelet therapy concluded that transfusion reduced mortality [37].

The PATCH trial explored the potential benefit of platelet transfusion in 190 patients treated with antiplatelet agents, mainly aspirin as monotherapy (149/190), and presenting supratentorial intracerebral haemorrhage with Glasgow Coma Scores ≥ 8 on admission and not requiring emergency neurosurgery [38]. Platelet transfusion was associated with a higher mortality and dependence at three months than the control group. One could argue that aspirin is not such an important risk factor of worse outcome and that platelet transfusion might not be beneficial because the absolute increase in intracerebral haemorrhage growth associated with antiplatelet therapy is not large enough to be meaningfully modified by platelet transfusion Likewise, negative results were obtained from observational studies of patients on APA therapy with intracerebral haemorrhage receiving platelet transfusion or not [39]. Patients treated with platelet transfusions had a higher risk of recourse to surgery, disability, and death. After matching with appropriate intracerebral haemorrhage score, transfusion was neither a significant predictor for poor outcome nor associated with any improvement. To date, this is the best evidence available, thus platelet transfusion should be avoided in case of non-traumatic ICH that does not require urgent neurosurgery in aspirin-treated patients presenting a Glasgow Coma Score ≥ 8 on admission. *In contrast, preoperative neutralisation of antiplatelet therapy is proposed in case of intracranial haemorrhage requiring urgent neurosurgery.* Indeed, the benefit of platelet transfusion to neutralise APAs comes from a randomised trial that included 366 patients treated with aspirin requiring emergent craniotomy [40]. Patients receiving platelet transfusion had less postoperative complications, disability, and mortality as compared to patients not transfused. Of note, in this trial, platelet transfusion was performed using previously frozen apheresis platelets, which are quite different from regular platelet concentrates. Indeed, freezing and thawing processes damage cryopreserved platelets. Approximately a 75% cell recovery is standard [41]. Nevertheless, when assayed using current parameters including morphology, flow cytometry for marker expression, as well as thrombin capacity, cryopreserved platelet product presents with a phenotype primed for haemostatic plug formation compared to a liquid-stored platelet [41].

Notably, the potential benefit of transfusion in patients presenting intracerebral haemorrhage with altered consciousness or in cases of treatment by P2Y_12_ receptor inhibitors has never been evaluated. *In such situations, it is proposed to discontinue antiplatelet therapy*.

Traumatic brain injury (TBI) is a specific class of ICH. The relationship between TBI outcome and pre-injury antiplatelet therapy is controversial. Especially, aspirin exposure is not always associated with progression of haemorrhage on Computer Tomography-scan, clinical deterioration, or mortality [19]. In contrast, pre-injury clopidogrel is associated with progression of haemorrhage, and increased risk for unfavourable long-term neurological outcomes. The meta-analysis of Marincowitz confirmed the relationship between clopidogrel and clinical deterioration or neurosurgical intervention, but no association between aspirin use and these outcomes in TBI patients [42].

Reversal of antiplatelet therapy in traumatic ICH is also controversial. It is not clear whether APA neutralisation effectively reduces progression of haemorrhage and improves outcome. The meta-analysis of studies assessing the impact of platelet transfusion in patients with TBI and preinjury antiplatelet therapy did not show any statistically significant benefit of platelet transfusion on mortality [43]. Notably, three studies showed significant evidence of harm. Nevertheless, the ten included studies included mostly aspirin-treated patients and were mainly retrospective, all small in size, with cohorts ranging from 66 to 328 patients, exposing to bias. Few data have specifically investigated the reversal of P2Y_12_ agents after ICH. Jehan assessed the effects of platelet transfusion in a prospective cohort of 243 patients with isolated TBI and ICH on preinjury P2Y_12_ inhibitor [44]. A total of 73.6% received platelet transfusion after admission. After controlling for confounders, platelet transfusion was associated with decreased risk of progression of ICH (Odd Ratio (OR): 0.68, *p* = 0.01), neurosurgical intervention (OR: 0.80, *p* = 0.03), and mortality (OR: 0.85, *p* = 0.04) on multivariate regression analysis after controlling for confounding factors. These results need to be confirmed but suggest that platelet transfusion is associated with improved outcomes in patients on preinjury P2Y_12_ inhibitors with traumatic ICH. Hence, and as stated in most guidelines, in case of intracranial haemorrhage requiring urgent neurosurgery, preoperative neutralisation of antiplatelet therapy is proposed. *In case of intracranial haemorrhage that does not require urgent neurosurgery, it is proposed to not transfuse platelets if the patient is on aspirin as monotherapy and presents a Glasgow Coma Score ≥ 8 on admission.*

### 3.3. Gastrointestinal Haemorrhage

Gastrointestinal bleeding is the most frequent situation associated with APAs. APAs increase both the severity of bleeding and the risk of rebleeding [45]. As in other severe haemorrhages, conventional haemostatic measures are sufficient to control the bleeding in most situations. International guidelines usually propose that aspirin is neither stopped nor neutralised. A cohort study compared patients with gastrointestinal bleeding who continued long-term aspirin after admission with those who discontinued it [46]. Patients who interrupted their treatment had fewer rebleeding events, but significantly more cardiovascular events and deaths. The efficacy of APA neutralisation in mitigating gastrointestinal bleeding has not been well evaluated, and the rare available data are retrospective, with major methodological limits, and did not show a benefit of transfusion [47,48]. As for other severe haemorrhages, guidelines propose *neutralisation of antiplatelet therapy in case of persistence of haemorrhage after failure of etiological and symptomatic treatments* [1,49]. Then, proton-pump inhibitor therapy is proposed to prevent rebleeding in patients who require single- or dual-antiplatelet therapy for a duration consistent with the ongoing need for antiplatelet therapy.

### 3.4. Non-Severe Haemorrhages

Non-severe haemorrhages are the most frequent. *Symptomatic treatment is proposed, without neutralisation of antiplatelet therapy*. Their management also includes re-evaluation of the indication for antiplatelet treatment. 

## 4. Place of Platelet Function Testing

The GIHP, as well as others [1,18,50], proposes to use platelet function testing (PFT) to identify platelet dysfunctions, no matter the causes (APA or other), when they are suspected on a clinical basis, in teams trained in and accustomed to the use of the tests.

Such a recommendation regarding PFT may look restrictive compared to the amount of information that these tests are expected to give. However, little data support the clinical benefit of using PFT for the management of bleeding events in patients treated or potentially treated with APA.

First, PFT might be useful to detect antiplatelet therapy and to guide bleeding management. Antiplatelet medication use is common among bleeding patients, but this information is not always available on admission: patients may be treated with APA but unconscious, mentally incapacitated, amnesic, or with variable therapeutic observance, or, in contrast, they may be not long-term-treated with APA but with a recent intake. When a PFT centred on a molecular target of an APA does not detect an APA effect, the result is consistent with distant last intake or non-observance, which consequently excludes the APA responsibility in the bleeding complication. However, many PFT are available, PFT are not interchangeable, they do not explore the same aspects of platelet function, their sensitivity varies, with remaining uncertainty regarding the ability of some of the PFT to detect subtle but clinically relevant platelet dysfunctions during bleeding [1,51]. Even if the intensity of platelet dysfunction and the severity of bleeding are relatively well correlated [1], few studies assessed the relationship between the level of platelet function alteration and associated bleeding severity, specifically in the perioperative setting. Moreover, thresholds corresponding to the degree of platelet inhibition or residual function associated with increased intensity of bleeding are unknown. In addition, platelet dysfunctions observed with PFT during severe bleeding are not specific of APA treatment as bleeding-induced haemostasis disturbances include platelet dysfunction by itself. Especially, platelet dysfunction is strikingly common after severe trauma, occurring in up to 45% of patients on admission, independently of APA treatment [52]. Impairment of platelet function was observed in 45% and 31% of patients in response to arachidonic acid and to ADP, respectively [53]. Isolated TBI is also accompanied with alterations of platelet ADP and thromboxane pathways, independent of haemorrhagic shock and linked to the severity of brain injury [54]. Therefore, PFT often fail to detect an APA effect during severe bleeding.

Second, PFT might be useful to guide platelet transfusion during treatment of bleeding complications. A few studies including patients with traumatic brain injury and a history of treatment with APAs have tried to use PFT to rationalise platelet transfusion: they proposed to transfuse platelets to patients with detectable platelet inhibition and not to patients with functional platelets according to PFT as they would be unlikely to benefit from platelet transfusion [40,55]. Moreover, platelet transfusion carries risks, including hypersensitivity side effects, transfusion-related acute lung injury, infection transmission, and immunomodulation. In the study including patients with acute intracerebral haemorrhage requiring craniectomy, platelet aggregation testing was used to guide transfusion of frozen platelets: patients who had received aspirin but were poorly affected by the APA according to the test were not transfused but had the same rate of postoperative haemorrhage, average postoperative haemorrhage volume, and mortality rate as patients who had not received aspirin [40]. This study supports the potential clinical interest of PFT for deciding whether or not to transfuse platelets to patients with intracerebral haemorrhage. Nevertheless, extrapolation of these results to other settings must be considered with caution and the exact role of PFT to guide platelet transfusion during bleeding has to be better established.

Third, PFT might be useful to monitor the reversal of APA by platelet transfusion during bleeding management and to guide the number of transfused platelets. In a prospective study of APA-treated patients with TBI, 51% and 67% of patients treated with aspirin or with clopidogrel respectively, did not experience reversal of platelet dysfunction after platelet transfusion, which suggests that additional platelet transfusion was required. Choi et al. found in a cohort of patients with traumatic brain injury and a history of antiplatelet therapy that platelet transfusion failed to reverse platelet dysfunction in 51% and 67% of patients taking aspirin or clopidogrel respectively, which again suggests that additional platelet transfusion was required [55]. Similarly, in a cohort of patients with TBI, Bachelani et al. reported reversal of aspirin-induced platelet dysfunction for approximately 65% of patients using VerifyNow^®^ [56]. Taylor et al. studied a cohort of patients requiring platelet transfusion for life-threatening haemorrhage or before emergent neurosurgery [57]. Transfusion of one unit of platelet concentrate per 10 kg reversed platelet dysfunction as assessed with VerifyNow^®^ for 12 out of the 13 patients on aspirin as monotherapy but did not reverse platelet dysfunction for patients on clopidogrel, indicating that the amount of transfused platelets was not sufficient to overcome platelet inhibition in the latter case.

## 5. Conclusions

The management of bleeding events per se relies on multiple therapies, including the reversal of antithrombotic drugs. In this regard, antiplatelet agents appear to play a mixed role in the genesis and maintenance of bleeding depending on the type of APA, the mechanism, and the site of the bleeding. Despite poor clinical evidence, platelet transfusion remains the principal option as an attempt to neutralise APA’s effect on primary haemostasis. Other therapeutic options (rFVIIa, desmopressin, specific antidotes) are either ineffective or poorly studied, or still under development. Platelet function tests are attractive but their impact on the therapeutic strategy and especially their place to guide platelet transfusion during bleeding remains to be validated.
